# Curious Cradles

**Published:** 1889-02

**Authors:** 


### CURIOUS CRADLES.

The Chinese have a queer institution which they call the winter cradle. It is shaped like an hour-glass, and stands on end. There is an opening above and below, and the waist, which is contracted, serves to keep the Celestial baby on his feet. Day after day little almond-shaped eyes peep over the top of this cradle, and little hands play with miniature dragons and other toys till the nurse puts in an appearance. Some of these winter cradles are made of wicker-work and are beautifully painted by Chinese women artists. It is almost impossible for one to be upset ; but now and then, when two are placed together and the occupants declare war and measure arms, two cradles roll over the floor to noises that "bring down the house." The Lapp baby very often has a snow cradle, for when the indulgent mother attends church she makes a hole in the snow outside and deposits the young Laplander therein. It is no uncommon sight to see a circle of these snow cradles in front of a Lapp chapel, and now and then a lot of fierce-looking dogs are on guard to keep off the wolves that might meditate a raid on the baby contingent. The Lapp cradle in material differs essentially from that used by the Bushman baby, whose mother digs a hole in the hot sand and chucks him therein in the shadow of some lonely bush. Sometimes the cradle is at hand in the shape of an ostrich nest, and now and then some of the feathers left by the mighty bird help to soften the nest of the future Bushman warrior. There is a tribe in the palm region of the Amazon that cradles the young in palm leaves. A single leaf turned up around the edges by some native process makes an excellent cradle, and now and then it is made to do service as a bath-tub. Strong cords are formed from the sinews of another species of palm, and by these this natural cradle is swung alongside a tree, and the wind rocks the little tot to sleep. Long ago the Amazonian mother discovered that it was not wise to leave a baby and cradle under a cocoa palm, for the mischievous monkeys delighted to drop the nuts downward with unerring precision. An older child is stationed near by to watch the baby during his sleep, and the chatter of the monkeys overhead is enough to cause a speedy migration. Patagonian babies are kept in cradles made of flat pieces of board. Two pieces of guanaco skin are so arranged across the table that the child is firmly fastened inside, and can be carried thus suspended from a saddle-bow without danger. In the rude huts of this people these cradles are hung hammock-wise to the rafters, and, amid the smoke that darkens everything, including his very nature, as it seems, the Patagonian infant passes the first stages of babyhood. When the village migrates, the cradle is swung from the saddle; and in swimming a stream it floats like a canoe on the surface, while the horse is almost entirely submerged. Sir Francis Head, who saw a good deal of Patagonian life years ago, leaves on record the statement that the Patagonian baby in its queer cradle is one of the best-natured representatives of the infant world. One would hardly go to Kaffirland for a fantastic cradle, and one almost as queer as it is fantastic. Yet he would find such a one there. The Kaffir baby, when he comes into the world, is put into a cradle or bag made of antelope skin, with the hair on. This baby castle, narrow toward the bottom, widens to within a few inches of the opening, when it again suddenly contracts. The skin is turned inward, giving the young Kaffir as soft a bed as some found in the cradles of royalty. Four long strips of antelope skin are attached to the cradle, and enable the mother to swing it on her back after a peculiar fashion.

